# Supplementary material for: HIVprotI: an integrated web based platform for prediction and design of HIV proteins inhibitors
Source: J Cheminform. 2018 Mar 9;10:12. doi: 10.1186/s13321-018-0266-y (PMC5845081; doi:10.1186/s13321-018-0266-y)
Supplement: Supplementary file 2 — Additional file 2. Source code of HIVProtI web server. [file 13321_2018_266_MOESM2_ESM.zip › HIVprotI_Source-code/hivprotI/weka-3-6-6/doc/weka/gui/beans/package-tree.html]

weka.gui.beans Class Hierarchy


---


|  |  |  |
| --- | --- | --- |
| **Overview** \| **Package** \| Class \| **Tree** \| **Deprecated** \| **Index** \| **Help** \| **Weka's home** | |  |
| **PREV**   **NEXT** | **FRAMES**    **NO FRAMES**     **All Classes** |  |


---


## Hierarchy For Package weka.gui.beans

**Package Hierarchies:**: All Packages

---

## Class Hierarchy

- java.lang.Object
  - weka.gui.beans.**BeanConnection** (implements java.io.Serializable)- weka.gui.beans.**BeanInstance** (implements java.io.Serializable)- java.awt.Component (implements java.awt.image.ImageObserver, java.awt.MenuContainer, java.io.Serializable)
        - java.awt.Container
          - javax.swing.JComponent (implements java.io.Serializable)
            - javax.swing.JPanel (implements javax.accessibility.Accessible)
              - weka.gui.beans.**AbstractDataSink** (implements weka.gui.beans.BeanCommon, weka.gui.beans.DataSink, weka.gui.beans.DataSourceListener, weka.gui.beans.InstanceListener, java.io.Serializable, weka.gui.beans.TestSetListener, weka.gui.beans.ThresholdDataListener, weka.gui.beans.TrainingSetListener, weka.gui.beans.Visible)
                - weka.gui.beans.**Saver** (implements weka.core.EnvironmentHandler, weka.gui.beans.WekaWrapper)
                - weka.gui.beans.**AbstractDataSource** (implements java.beans.beancontext.BeanContextChild, weka.gui.beans.DataSource, java.io.Serializable, weka.gui.beans.Visible)
                  - weka.gui.beans.**Loader** (implements weka.gui.beans.BeanCommon, weka.core.EnvironmentHandler, weka.gui.beans.EventConstraints, weka.gui.beans.Startable, weka.gui.beans.StructureProducer, weka.gui.beans.WekaWrapper)
                  - weka.gui.beans.**AbstractEvaluator** (implements weka.gui.beans.BeanCommon, java.io.Serializable, weka.gui.beans.Visible)
                    - weka.gui.beans.**ClassifierPerformanceEvaluator** (implements weka.gui.beans.BatchClassifierListener, weka.gui.beans.EventConstraints, java.io.Serializable, weka.gui.beans.UserRequestAcceptor)- weka.gui.beans.**ClustererPerformanceEvaluator** (implements weka.gui.beans.BatchClustererListener, weka.gui.beans.EventConstraints, java.io.Serializable, weka.gui.beans.UserRequestAcceptor)- weka.gui.beans.**IncrementalClassifierEvaluator** (implements weka.gui.beans.EventConstraints, weka.gui.beans.IncrementalClassifierListener)
                    - weka.gui.beans.**AbstractTestSetProducer** (implements weka.gui.beans.BeanCommon, java.io.Serializable, weka.gui.beans.TestSetProducer, weka.gui.beans.Visible)
                      - weka.gui.beans.**TestSetMaker** (implements weka.gui.beans.DataSourceListener, weka.gui.beans.EventConstraints, java.io.Serializable)
                      - weka.gui.beans.**AbstractTrainAndTestSetProducer** (implements weka.gui.beans.BeanCommon, weka.gui.beans.DataSourceListener, java.io.Serializable, weka.gui.beans.TestSetProducer, weka.gui.beans.TrainingSetProducer, weka.gui.beans.Visible)
                        - weka.gui.beans.**CrossValidationFoldMaker** (implements weka.gui.beans.DataSourceListener, weka.gui.beans.EventConstraints, java.io.Serializable, weka.gui.beans.TestSetListener, weka.gui.beans.TrainingSetListener, weka.gui.beans.UserRequestAcceptor)- weka.gui.beans.**TrainTestSplitMaker** (implements weka.gui.beans.DataSourceListener, weka.gui.beans.EventConstraints, java.io.Serializable, weka.gui.beans.TestSetListener, weka.gui.beans.TrainingSetListener, weka.gui.beans.UserRequestAcceptor)
                        - weka.gui.beans.**AbstractTrainingSetProducer** (implements weka.gui.beans.BeanCommon, java.io.Serializable, weka.gui.beans.TrainingSetProducer, weka.gui.beans.Visible)
                          - weka.gui.beans.**TrainingSetMaker** (implements weka.gui.beans.DataSourceListener, weka.gui.beans.EventConstraints, java.io.Serializable)
                          - weka.gui.beans.**Associator** (implements weka.gui.beans.BeanCommon, weka.gui.beans.DataSourceListener, weka.gui.beans.EventConstraints, java.io.Serializable, weka.gui.beans.TrainingSetListener, weka.gui.beans.UserRequestAcceptor, weka.gui.beans.Visible, weka.gui.beans.WekaWrapper)- weka.gui.beans.**AssociatorCustomizer** (implements java.beans.Customizer, weka.gui.beans.CustomizerCloseRequester)- weka.gui.beans.**BeanVisual**- weka.gui.beans.**ClassAssigner** (implements weka.gui.beans.BeanCommon, weka.gui.beans.DataSource, weka.gui.beans.DataSourceListener, weka.gui.beans.EventConstraints, weka.gui.beans.InstanceListener, java.io.Serializable, weka.gui.beans.StructureProducer, weka.gui.beans.TestSetListener, weka.gui.beans.TestSetProducer, weka.gui.beans.TrainingSetListener, weka.gui.beans.TrainingSetProducer, weka.gui.beans.Visible)- weka.gui.beans.**ClassAssignerCustomizer** (implements java.beans.Customizer, weka.gui.beans.CustomizerCloseRequester, weka.gui.beans.CustomizerClosingListener, weka.gui.beans.DataFormatListener)- weka.gui.beans.**Classifier** (implements weka.gui.beans.BeanCommon, weka.gui.beans.EventConstraints, weka.gui.beans.InstanceListener, java.io.Serializable, weka.gui.beans.TestSetListener, weka.gui.beans.TrainingSetListener, weka.gui.beans.UserRequestAcceptor, weka.gui.beans.Visible, weka.gui.beans.WekaWrapper)- weka.gui.beans.**ClassifierCustomizer** (implements java.beans.Customizer, weka.gui.beans.CustomizerCloseRequester, weka.gui.beans.CustomizerClosingListener)- weka.gui.beans.**ClassValuePicker** (implements weka.gui.beans.BeanCommon, weka.gui.beans.DataSourceListener, weka.gui.beans.EventConstraints, java.io.Serializable, weka.gui.beans.StructureProducer, weka.gui.beans.Visible)- weka.gui.beans.**ClassValuePickerCustomizer** (implements java.beans.Customizer, weka.gui.beans.CustomizerCloseRequester, weka.gui.beans.CustomizerClosingListener)- weka.gui.beans.**Clusterer** (implements weka.gui.beans.BeanCommon, weka.gui.beans.EventConstraints, weka.gui.beans.TestSetListener, weka.gui.beans.TrainingSetListener, weka.gui.beans.UserRequestAcceptor, weka.gui.beans.Visible, weka.gui.beans.WekaWrapper)- weka.gui.beans.**ClustererCustomizer** (implements java.beans.Customizer, weka.gui.beans.CustomizerCloseRequester)- weka.gui.beans.**CostBenefitAnalysis** (implements weka.gui.beans.BeanCommon, java.beans.beancontext.BeanContextChild, java.io.Serializable, weka.gui.beans.ThresholdDataListener, weka.gui.beans.UserRequestAcceptor, weka.gui.beans.Visible)- weka.gui.beans.**CrossValidationFoldMakerCustomizer** (implements java.beans.Customizer)- weka.gui.beans.**DataVisualizer** (implements java.beans.beancontext.BeanContextChild, weka.gui.beans.DataSourceListener, java.io.Serializable, weka.gui.beans.TestSetListener, weka.gui.beans.TrainingSetListener, weka.gui.beans.UserRequestAcceptor, weka.gui.beans.Visible)
                                                      - weka.gui.beans.**AttributeSummarizer**- weka.gui.beans.**ScatterPlotMatrix**
                                                      - weka.gui.beans.**Filter** (implements weka.gui.beans.BeanCommon, weka.gui.beans.DataSource, weka.gui.beans.DataSourceListener, weka.gui.beans.EventConstraints, weka.gui.beans.InstanceListener, java.io.Serializable, weka.gui.beans.TestSetListener, weka.gui.beans.TestSetProducer, weka.gui.beans.TrainingSetListener, weka.gui.beans.TrainingSetProducer, weka.gui.beans.UserRequestAcceptor, weka.gui.beans.Visible, weka.gui.beans.WekaWrapper)- weka.gui.beans.**FilterCustomizer** (implements java.beans.Customizer, weka.gui.beans.CustomizerCloseRequester)- weka.gui.beans.**GraphViewer** (implements java.beans.beancontext.BeanContextChild, weka.gui.beans.GraphListener, java.io.Serializable, weka.gui.beans.UserRequestAcceptor, weka.gui.beans.Visible)- weka.gui.beans.**IncrementalClassifierEvaluatorCustomizer** (implements java.beans.Customizer)- weka.gui.beans.**InstanceStreamToBatchMaker** (implements weka.gui.beans.BeanCommon, weka.gui.beans.DataSource, weka.gui.beans.EventConstraints, weka.gui.beans.InstanceListener, weka.gui.beans.Visible)- weka.gui.beans.**KnowledgeFlowApp** (implements java.beans.PropertyChangeListener)- weka.gui.beans.**LoaderCustomizer** (implements java.beans.Customizer, weka.gui.beans.CustomizerCloseRequester)- weka.gui.beans.**LogPanel** (implements weka.gui.Logger)- weka.gui.beans.**MetaBean** (implements weka.gui.beans.BeanCommon, weka.gui.beans.EventConstraints, java.io.Serializable, weka.gui.beans.UserRequestAcceptor, weka.gui.beans.Visible)- weka.gui.beans.**ModelPerformanceChart** (implements java.beans.beancontext.BeanContextChild, java.io.Serializable, weka.gui.beans.ThresholdDataListener, weka.gui.beans.UserRequestAcceptor, weka.gui.beans.Visible, weka.gui.beans.VisualizableErrorListener)- weka.gui.beans.**PredictionAppender** (implements weka.gui.beans.BatchClassifierListener, weka.gui.beans.BatchClustererListener, weka.gui.beans.BeanCommon, weka.gui.beans.DataSource, weka.gui.beans.EventConstraints, weka.gui.beans.IncrementalClassifierListener, java.io.Serializable, weka.gui.beans.TestSetProducer, weka.gui.beans.TrainingSetProducer, weka.gui.beans.Visible)- weka.gui.beans.**PredictionAppenderCustomizer** (implements java.beans.Customizer)- weka.gui.beans.**SaverCustomizer** (implements java.beans.Customizer, weka.gui.beans.CustomizerCloseRequester)- weka.gui.beans.**SerializedModelSaver** (implements weka.gui.beans.BatchClassifierListener, weka.gui.beans.BatchClustererListener, weka.gui.beans.BeanCommon, weka.core.EnvironmentHandler, weka.gui.beans.IncrementalClassifierListener, java.io.Serializable, weka.gui.beans.Visible)- weka.gui.beans.**SerializedModelSaverCustomizer** (implements java.beans.Customizer, weka.gui.beans.CustomizerCloseRequester)- weka.gui.beans.**StripChart** (implements weka.gui.beans.BeanCommon, weka.gui.beans.ChartListener, weka.gui.beans.InstanceListener, weka.gui.beans.UserRequestAcceptor, weka.gui.beans.Visible)- weka.gui.beans.**StripChartCustomizer** (implements java.beans.Customizer)- weka.gui.beans.**TextViewer** (implements weka.gui.beans.BeanCommon, java.beans.beancontext.BeanContextChild, weka.gui.beans.DataSourceListener, weka.gui.beans.EventConstraints, weka.gui.beans.TestSetListener, weka.gui.beans.TextListener, weka.gui.beans.TrainingSetListener, weka.gui.beans.UserRequestAcceptor, weka.gui.beans.Visible)- weka.gui.beans.**TrainTestSplitMakerCustomizer** (implements java.beans.Customizer)
        - java.util.EventObject (implements java.io.Serializable)
          - weka.gui.beans.**BatchClassifierEvent**- weka.gui.beans.**BatchClustererEvent**- weka.gui.beans.**ChartEvent**- weka.gui.beans.**DataSetEvent**- weka.gui.beans.**GraphEvent**- weka.gui.beans.**IncrementalClassifierEvent**- weka.gui.beans.**InstanceEvent**- weka.gui.beans.**TestSetEvent**- weka.gui.beans.**TextEvent**- weka.gui.beans.**ThresholdDataEvent**- weka.gui.beans.**TrainingSetEvent**- weka.gui.beans.**VisualizableErrorEvent**
          - weka.gui.beans.**FlowRunner** (implements weka.core.RevisionHandler)- weka.gui.beans.**FlowRunner.SimpleLogger** (implements weka.gui.Logger)- weka.gui.beans.**KnowledgeFlow**- weka.gui.beans.**Messages**- java.beans.SimpleBeanInfo (implements java.beans.BeanInfo)
                    - weka.gui.beans.**AbstractDataSinkBeanInfo**
                      - weka.gui.beans.**SaverBeanInfo**
                      - weka.gui.beans.**AbstractDataSourceBeanInfo**
                        - weka.gui.beans.**LoaderBeanInfo**
                        - weka.gui.beans.**AbstractTestSetProducerBeanInfo**
                          - weka.gui.beans.**TestSetMakerBeanInfo**
                          - weka.gui.beans.**AbstractTrainAndTestSetProducerBeanInfo**
                            - weka.gui.beans.**CrossValidationFoldMakerBeanInfo**- weka.gui.beans.**TrainTestSplitMakerBeanInfo**
                            - weka.gui.beans.**AbstractTrainingSetProducerBeanInfo**
                              - weka.gui.beans.**TrainingSetMakerBeanInfo**
                              - weka.gui.beans.**AssociatorBeanInfo**- weka.gui.beans.**AttributeSummarizerBeanInfo**- weka.gui.beans.**ClassAssignerBeanInfo**- weka.gui.beans.**ClassifierBeanInfo**- weka.gui.beans.**ClassifierPerformanceEvaluatorBeanInfo**- weka.gui.beans.**ClassValuePickerBeanInfo**- weka.gui.beans.**ClustererBeanInfo**- weka.gui.beans.**ClustererPerformanceEvaluatorBeanInfo**- weka.gui.beans.**CostBenefitAnalysisBeanInfo**- weka.gui.beans.**DataVisualizerBeanInfo**- weka.gui.beans.**FilterBeanInfo**- weka.gui.beans.**GraphViewerBeanInfo**- weka.gui.beans.**IncrementalClassifierEvaluatorBeanInfo**- weka.gui.beans.**InstanceStreamToBatchMakerBeanInfo**- weka.gui.beans.**ModelPerformanceChartBeanInfo**- weka.gui.beans.**PredictionAppenderBeanInfo**- weka.gui.beans.**ScatterPlotMatrixBeanInfo**- weka.gui.beans.**SerializedModelSaverBeanInfo**- weka.gui.beans.**StripChartBeanInfo**- weka.gui.beans.**TextViewerBeanInfo**

## Interface Hierarchy

- weka.gui.beans.**BeanCommon**- weka.gui.beans.**ConnectionNotificationConsumer**- weka.gui.beans.**CustomizerCloseRequester**- weka.gui.beans.**CustomizerClosingListener**- weka.gui.beans.**DataFormatListener**- weka.gui.beans.**DataSink**- weka.gui.beans.**DataSource**- weka.gui.beans.**EventConstraints**- java.util.EventListener
                  - weka.gui.beans.**BatchClassifierListener**- weka.gui.beans.**BatchClustererListener**- weka.gui.beans.**ChartListener**- weka.gui.beans.**DataSourceListener**- weka.gui.beans.**GraphListener**- weka.gui.beans.**IncrementalClassifierListener**- weka.gui.beans.**InstanceListener**- weka.gui.beans.**StartUpListener**- weka.gui.beans.**TestSetListener**- weka.gui.beans.**TextListener**- weka.gui.beans.**ThresholdDataListener**- weka.gui.beans.**TrainingSetListener**- weka.gui.beans.**VisualizableErrorListener**
                  - weka.gui.beans.**LogWriter**- weka.gui.beans.**Startable**- weka.gui.beans.**StructureProducer**- weka.gui.beans.**TestSetProducer**- weka.gui.beans.**TrainingSetProducer**- weka.gui.beans.**UserRequestAcceptor**- weka.gui.beans.**Visible**- weka.gui.beans.**WekaWrapper**

---


|  |  |  |
| --- | --- | --- |
| **Overview** \| **Package** \| Class \| **Tree** \| **Deprecated** \| **Index** \| **Help** \| **Weka's home** | |  |
| **PREV**   **NEXT** | **FRAMES**    **NO FRAMES**     **All Classes** |  |


---
